# A Novel Rabbit Anti-Myoglobin Monoclonal Antibody’s Potential Application in Rhabdomyolysis Associated Acute Kidney Injury

**DOI:** 10.3390/ijms24097822

**Published:** 2023-04-25

**Authors:** Xinyue Wang, Ou Qiao, Lu Han, Ning Li, Yanhua Gong

**Affiliations:** 1 Institute of Disaster and Emergency Medicine, Medical College, Tianjin University, Tianjin 300072, China; wxy435014@tju.edu.cn (X.W.); qiaoou@tju.edu.cn (O.Q.); stu_hanlu@tju.edu.cn (L.H.); 2 Tianjin Key Laboratory of Disaster Medicine Technology, Tianjin 300072, China

**Keywords:** myoglobin, rabbit monoclonal antibody, recombinant antibody, rhabdomyolysis, hybridoma cells

## Abstract

Myoglobin (Mb) is the main constituent of vertebrate skeletal muscle and myocardium and plays an essential role in oxygen binding, storage, transport, and earliest disease diagnosis. This study focuses on preparing the novel recombinant rabbit anti-Mb monoclonal antibody and applying it to a diagnosis of Mb deposition in rhabdomyolysis-associated acute kidney injury (RM-AKI). The full-length coding sequence of rat Mb was cloned and expressed, and the high-quality and titer rabbit anti-Mb polyclonal antibodies were produced by the immunogen His-Mb fusion protein. A new hybridoma cell was obtained by hybridoma screening technology. With the help of DNA sequencing and a molecular clonal, anti-Mb monoclonal antibody heavy and light chains expression plasmid was constructed. Finally, the recombinant rabbit anti-Mb monoclonal antibody with extraordinarily high affinity (K_D_ = 1.21 pM) was obtained. Meanwhile, it had broad species reactivity (mouse, rat, human, and horse) and good tissue specificity (skeletal muscle and myocardium). It also had a very good performance in western blotting, immunohistochemistry, and immunofluorescence assay to detect the Mb level in the kidney, myocardium, and skeletal muscle of RM-AKI. This study will be significantly helpful for Mb-associated disease diagnosis, and pathogenesis exploration, and further may act as a neutralizing antibody for disease treatment.

## 1. Introduction

Myoglobin (Mb) is a small monomeric cytoplasmic hemoprotein with a relative molecular weight of 17.8 kDa [1]. It is mainly expressed in vertebrate cardiac and skeletal muscle where it plays an essential role in oxygen binding, storage, and transport to the mitochondria [2,3]. Previous studies have shown the ratio of Mb (mg) to tissue wet weight (g) is about 2.5 in the myocardium, and the value in the skeletal muscle is about 4.0 [4]. Mb is composed of globin (α-globin chain and β-globin chain) and a heme group [5]. Normally, Mb is found in the striated muscles and cardiac muscles of vertebrates [6]. However, if a lot of Mb appears in the blood, urine, or organs such as the kidneys, this is very dangerous.

Rhabdomyolysis (RM) is a potentially life-threatening syndrome and is characterized by the damage to skeletal muscle and the release of intracellular breakdown products (mainly including Mb) into the blood circulation [7,8]. The triggers for rhabdomyolysis are extensive and include crush injuries, trauma, certain infections, drugs, alcohol, and toxins [9]. Mb acts as a key factor in the pathophysiology of rhabdomyolysis. This protein is the main constituent of skeletal muscle that contributes to the acute kidney injury (AKI) which is a dangerous complication of severe rhabdomyolysis [10]. Current research indicated that Mb causes AKI by three mechanisms. The first mechanism is the Mb toxicity of tubular obstruction. Second, the oxidant injury is caused by iron dissociation in the Mb and free radicals’ release. The final mechanism is due to the lipid peroxidation caused by Mb [10].

Antibodies are usually applied in both diagnostics and basic research. Medical laboratory testing has important guiding significance for physicians’ diagnosis and treatment and avoids redundant or ineffective testing. The laboratory testing for Mb is for myocardial infarction or rhabdomyolysis. Mb is one of the earliest sensitive acute myocardial infarction biomarkers of which the concentration rises in the blood 30 min immediately after the beginning of the event [1,11]. Meanwhile, Mb is an important earliest biomarker for ischemic heart disease [12]. With regards to clinical rhabdomyolysis, some experts believe that myoglobin is not a prognostic and diagnostic criterion [13]. However, it is still a routine biochemical indicator with blood urea nitrogen (BUN), serum creatinine (Scr), and creatine kinase (CK) for hospital testing of rhabdomyolysis patients. In addition, the detection of Mb is more expensive than other conventional biochemical indicators, so the development of high-affinity Mb antibodies is expected to reduce the cost of clinical detection. Meanwhile, in basic research on laboratory animals, is still a key biomarker for kidney injury of RM-AKI or crush syndrome-AKI (CS-AKI) [10,14,15].

Antibody detection of Mb depends on the specificity and affinity of the antibody. Monoclonal antibody only targets a specific epitope that has the advantages of high purity and strong specificity. It has been widely used in various biomedical sciences fields and promoted many discipline developments. Especially recombinant rabbit monoclonal antibodies, has its outstanding advantages, including better specificity and sensitivity [16], high batch-to-batch consistency and reproducibility, animal origin-free formulations, long-term security of supply [17], and broader immunoreactivity to diverse targets due to larger rabbit immune repertoire [18]. Rabbit monoclonal antibodies against Mb have been successfully prepared and commercially sold for laboratory research [19,20,21]. However, the immunogen is truncated human Mb (P02144) of commercial Rabbit monoclonal antibody, not Rat Mb (Q9QZ76). Moreover, the species reactivity and applications are limited for the commercial antibodies (ABclonal, Cat: A11368; CST, Cat: #25919; Abcam, Cambridge, UK, Cat: ab242767). In addition, the affinity is not very high (equilibrium dissociation constant (K_D_) is about 10^−10^ M, Abcam, Cat: ab77232). Unfortunately, to date, high affinity, broad species reactivity, and multifaceted applications of recombinant rabbit anti-Mb monoclonal antibody that is prepared for rat Mb immunogen have not been reported.

Therefore, in this study, the novel recombinant rabbit anti-rat Mb monoclonal antibodies prepared using eukaryotic expression of exogenous rat Mb gene, animal immunization, hybridoma screening, heavy chain and light chain gene sequencing and mass ratio optimization of transient transfection, recombinant antibody expression and purification, and other technologies. The recombinant rabbit anti-Mb monoclonal antibody with extraordinarily high affinity (K_D_ = 1.21 pM), broad species reactivity (mouse, rat, human, and horse), and good tissue specificity (skeletal muscle and myocardium). At least, we applied the recombinant rabbit anti-Mb monoclonal antibodies to diagnose Mb deposition in the kidney, myocardium, skeletal muscle tissue, and blood of RM-AKI disease by western blotting (WB), immunohistochemistry, and immunofluorescence. In the future, this novel monoclonal antibody may act as a neutralizing antibody for the treatment of RM-related diseases.

## 2. Results

### 2.1. Purification of Rat Mb Recombinant Protein and Production of Plasma-Derived Rabbit Anti-Mb Polyclonal Antibodies

**Figure 1:**
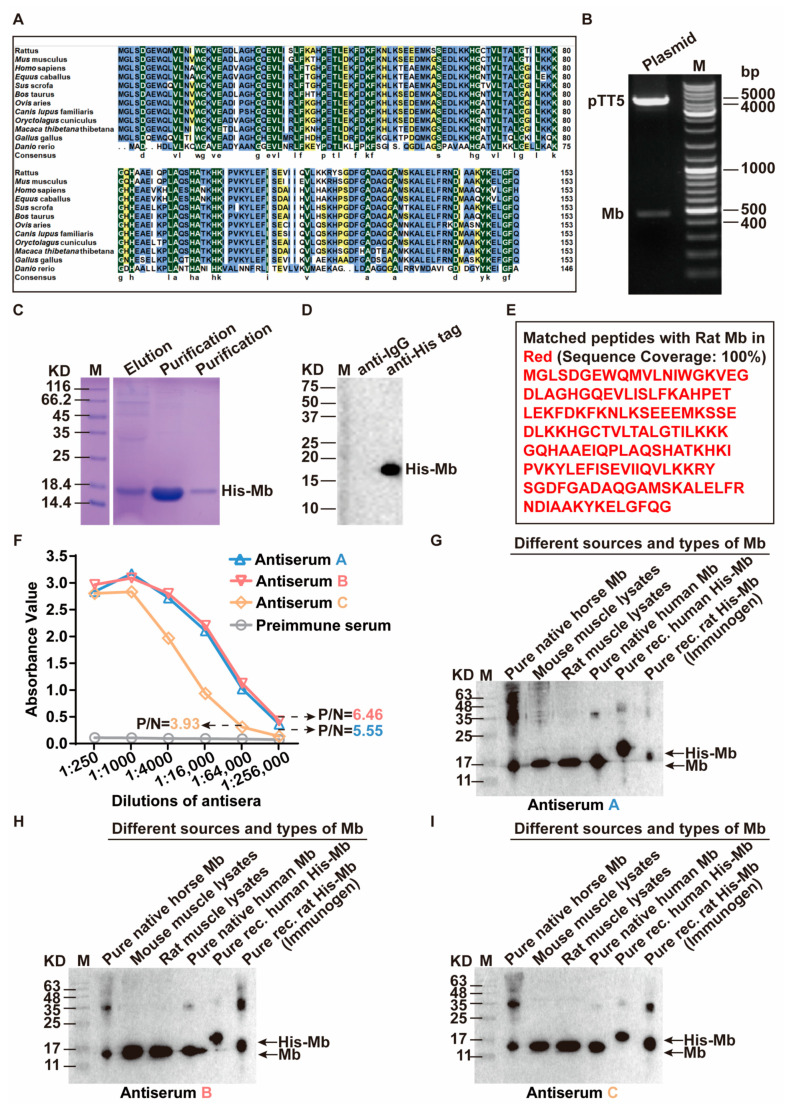
Preparation of immunogen His-Mb protein and generation of plasma-derived rabbit anti-Mb polyclonal antibodies. (**A**) The alignment of Mb protein amino acids sequence in different species (*Rattus*, *Mus*, *Homo*, *Equus*, *Sus*, *Bos*, *Ovis*, *Canis*, *Oryctolagus*, *Macaca*, *Gallus*, and *Danio*). (**B**) Double enzyme digestion identification of recombinant pTT5-Mb plasmid by EcoRI and BamHI. Lane 1: pTT5-Mb plasmid; Lane 2: M is DNA ladder (O’GeneRuler DNA Ladder Mix 100–10,000 bp). (**C**) SDS-PAGE and Coomassie brilliant blue staining analysis of purified His-Mb protein. Lane 1: M represents protein marker (14.4–116 kDa); Lane 2: The supernatant of HEK-293F cells containing His-Mb protein passes through the Ni-NTA affinity chromatography column; Lane 3–4: Purified His-Mb protein. (**D**) Immunoprecipitation assays verified the His-Mb protein. Lane 1: M represents protein marker (10–250 kDa); Lane 2: Recombinant rabbit IgG (Isotype Control); Lane 3: Anti-His antibody. (**E**) Mass spectrometry analysis of purified His-Mb protein. (**F**) The comparison of antibody titers in three rabbits (Rabbit A, B, C) immunized with His-Mb protein that evaluated by indirect ELISA. Red, blue, and orange represent positive antiserum that contains plasma-derived anti-Mb polyclonal antibodies. Grey represents pre-immune negative serum. Antiserum was serially diluted fourfold from 1:250 to 1:256,000. (**G**–**I**) WB analysis for species reactivity of plasma-derived anti-Mb polyclonal antibodies of Rabbit A, Rabbit B, and Rabbit C. Lane 1: M is protein marker (11–245 kDa); Lane 2: Pure native horse Mb (Beijing Solarbio Science & Technology Co., Ltd., Beijing, China, #M8090); Lane 3: Mouse muscle lysates (skeletal muscle lysis mixture of mice); Lane 4: Rat muscle lysates (skeletal muscle lysis mixture of rats); Lane 5: Pure native human Mb (Abcam, #ab96036); Lane 6: Pure recombinant human His-Mb (OkayBio, #K2916); Lane 7: Pure recombinant rat His-Mb protein (immunogen). The protein amounts of pure native horse Mb, mouse skeletal muscle lysate (mixture), rat skeletal muscle lysate (mixture), pure native human Mb, pure recombinant human Mb, and immunogen (rat His-Mb) are 5 µg (pure protein), 50 µg (total protein), 50 µg (total protein), 0.66 µg (pure protein), 0.264 µg (pure protein), and 0.21 µg (pure protein), respectively.

To elucidate the evolutionary characteristics of Mb among species, Mb amino acid sequences from different species (*Rattus*, *Mus*, *Homo*, *Equus*, *Sus*, *Bos*, *Ovis*, *Canis*, *Oryctolagus*, *Macaca*, *Gallus*, and *Danio*) were downloaded and sequenced. Results showed that the amino acid sequence identity of all the above species was about 85.98%. The identity and similarity in rats (*Rattus*) and humans (*Homo*) were about 83.12% and 91.6%, respectively (Figure 1A), and the sequence was relatively conserved. Given the high consistency of Mb genes in humans, rats, and mice, and the fact that our animal experiments were mainly focused on rats, we chose the Mb amino acid sequence of *Rattus norvegicus* as an immunogen considering subsequent applications.

The full-length rat Mb gene was synthesized and cloned into the pTT5 vector to construct the pTT5-Mb eukaryotic expression plasmid. Then, it was transformed into the Stbl3 competent cells for overnight culture on the drawing board and colonies. We extracted the plasmid from the positive colonies and identified it by double digestion experiment (Figure 1B) and sequencing comparison. The result of sequencing was as high as 100% similar to the rat Mb gene (gene identification 59,108/accession number NM_021588.2).

LipofectamineTM 2000 was used to transfect the correct pTT5-Mb plasmid to HEK-293F cells. The recombinant His-Mb fusion protein existed in the supernatant and was purified by the Ni-NTA affinity chromatography column. The purity of His-Mb protein was about 95% by SDS-PAGE and Coomassie staining analysis (Figure 1C). The correctness of recombinant His-Mb protein was verified by immunoprecipitation (IP) (Figure 1D) and mass spectrometry analysis (Figure 1E). Finally, we obtained sufficient amounts and purity of the immunogen His-Mb protein.

For animal immunization experiments, according to the method, the effective titer of antibody is that the ratio of positive serum to negative serum is greater than 2.1 [22,23,24]. The results showed that the anti-Mb seroprevalence was 100% and the strongest antiserum titer of Rabbit B was greater than 1:256,000 (Figure 1F).

Next, we performed WB to verify the species reactivity of plasma-derived rabbit anti-Mb polyclonal antibodies. As shown in Figure 1G–I, the anti-rat Mb serum from all rabbits could interact with Mb protein in different species and types (such as horse, mouse, rat, and human; native and recombinant). Therefore, based on the antiserum titer and species reactivity, we selected the anti-Mb polyclonal antibodies produced by Rabbit B for further antibody verification.

**Figure 2:**
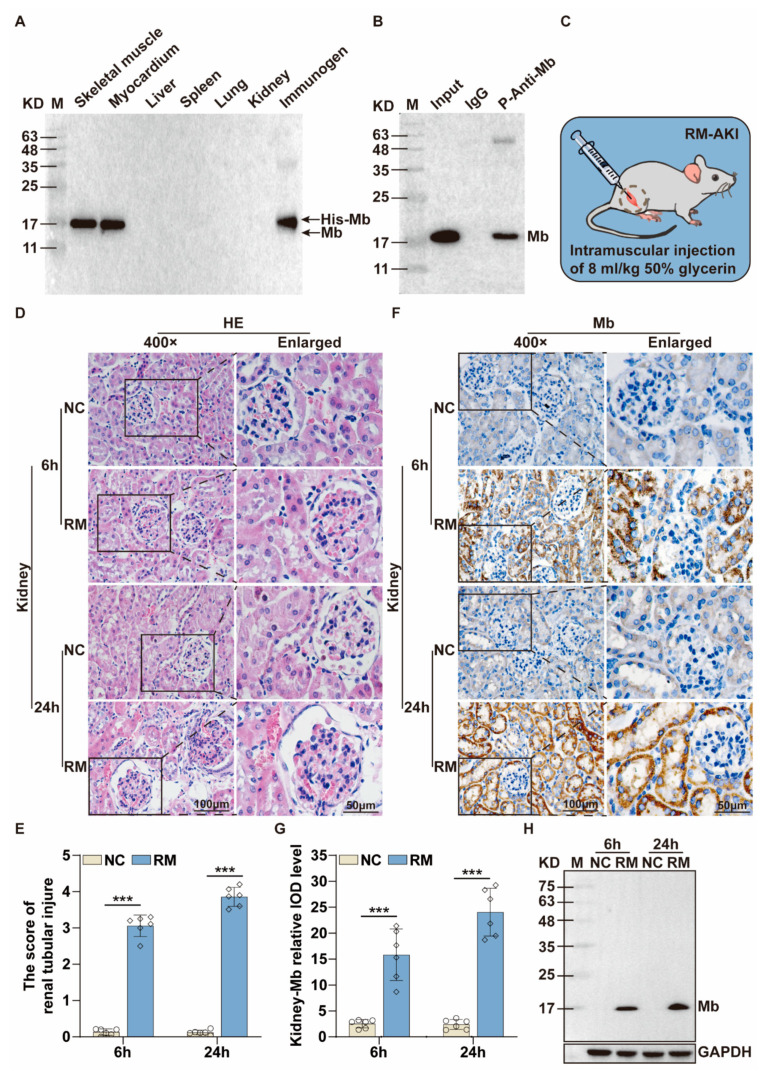
Characterization of plasma-derived rabbit anti-Mb polyclonal antibodies from Rabbit B induced by His-Mb protein. (**A**) The Mb protein expression in skeletal muscle, myocardium, liver, spleen, lung, and kidney of normal rats was detected by WB using antiserum from Rabbit B. Immunogen His-Mb protein acted as a positive control. (**B**) Immunoprecipitation of the plasma-derived rabbit anti-Mb polyclonal antibodies with rat skeletal muscle protein. IgG antibody acted as a negative control. (**C**) Schematic drawing of mice RM-AKI model preparation. (**D**) HE staining was used to analyze the kidney injury of RM-AKI mice at 6 h and 24 h (×400). (**E**) Renal tubular injury score was calculated from tubular dilation, cast formation, and tubular necrosis. Score 0 represented normal, and score 5 represented the most severe injury. (**F**) Immunohistochemistry staining detected the Mb protein distribution in the kidney tissue sections of RM-AKI mice with plasma-derived rabbit anti-Mb polyclonal antibodies (×400). (**G**) The integrated optical density (IOD) of Mb protein in each group (*n* = 6; ***, *p* < 0.001). (**H**) WB assays detected the changes in Mb protein expression in the kidney tissue of RM-AKI mice with plasma-derived rabbit anti-Mb polyclonal antibodies.

Moreover, we also conducted WB to detect tissue specificity of plasma-derived anti-Mb polyclonal antibodies from Rabbit B with the rat tissues. The results showed that these anti-Mb polyclonal antibodies could specifically recognize Mb protein in skeletal muscle and myocardium, but not in the liver, spleen, lung, and kidney of normal rats (Figure 2A). Meanwhile, immunoprecipitation results showed that these polyclonal antibodies had a binding affinity with rat skeletal muscle protein (Figure 2B). The above results showed that plasma-derived rabbit anti-Mb polyclonal antibodies had good specificity and affinity.

To further verify the effect of these polyclonal antibodies in the kidney tissues of RM-AKI, we established the mice RM-AKI model. The concentration of CK, Mb, BUN, and Scr in the blood serum of model mice were increased after injecting glycerin for 6 h or 24 h, respectively (Figure S1A–D). The mRNA expression level of kidney injury molecule-1 (KIM-1) and neutrophil gelatinase-associated lipocalin (NGAL) in the kidney were also increased in the RM-AKI group (Figure S1E,F). Meanwhile, the Hematoxylin-Eosin (HE) staining results showed amounts of red blood cell accumulation in the capillaries, glomerulus swelling, and tubular injury (Figure 2D). Tubular injury score was about 3 and 4 points at 6 h and 24 h in the RM-AKI group, respectively (Figure 2E). These results indicated that we successfully constructed the mice RM-AKI model. Immunohistochemistry staining results showed that Mb protein only existed in the kidney of the RM-AKI group, and the cumulative amount of Mb protein in kidney tissue at 24 h was higher than that at 6 h (Figure 2F,G). WB results were consistent with immunohistochemistry staining (Figure 2H). In addition, Mb protein was specifically deposited in the renal tubules of the RM-AKI group (Figure 2F). Overall, the recombinant His-Mb fusion protein had antigenicity and immunogenicity for antibody preparation, and the plasma-derived rabbit anti-Mb polyclonal antibodies from Rabbit B were able to specifically recognize and react with Mb protein. 

### 2.2. Screening and Characterization of Hybridoma-Derived Rabbit Anti-Mb Monoclonal Antibody

**Figure 3:**
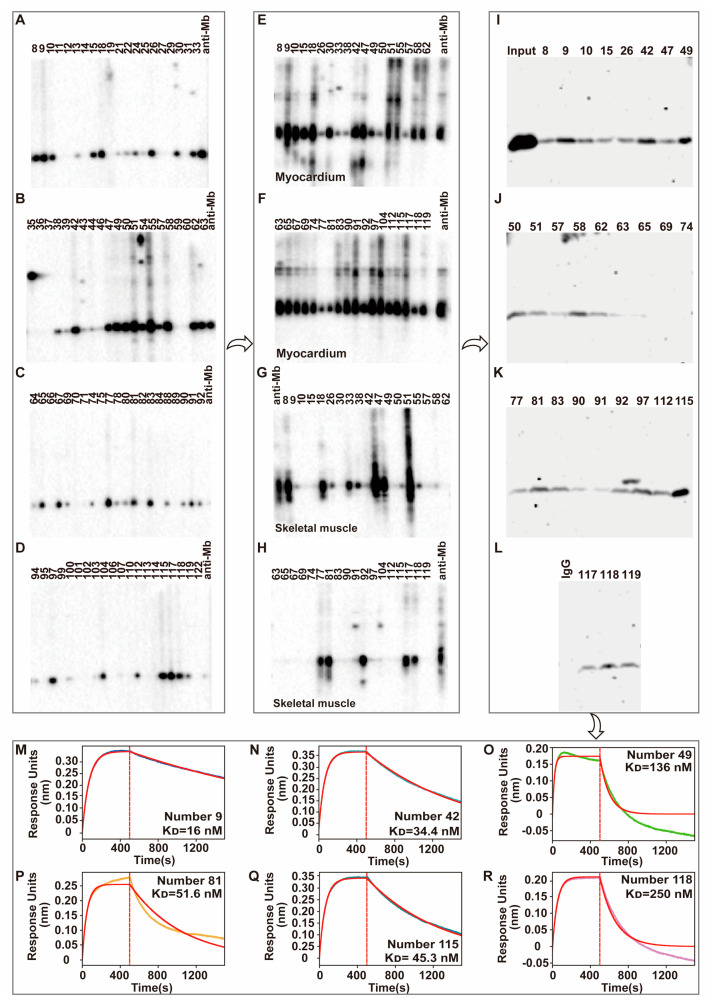
Screening and characterization of hybridoma-derived rabbit anti-Mb monoclonal antibody. (**A**–**D**) WB analysis of the reactivity of 82 hybridoma supernatants with immunogen His-Mb protein. Commercial anti-Mb antibodies acted as a positive control. (**E**,**F**) WB analysis of the reactivity of 36 positive hybridoma supernatants (screening from (**A**–**D**)) with mouse myocardium tissues protein. Commercial anti-Mb antibodies acted as a positive control. (**G**,**H**) WB analysis of the reactivity of 36 positive hybridoma supernatants (screening from (**A**–**D**)) with human skeletal muscle protein. Commercial anti-Mb antibodies acted as a positive control. (**I**–**L**) Immunoprecipitation analysis of 29 positive hybridoma supernatants (screening from (**A**–**F**)) with rat skeletal muscle. IgG antibody acted as a negative control. (**M**–**R**) Fortebio kinetic characterization assay analysis of the binding affinity of monoclonal antibody in hybridoma supernatants with His-Mb protein. The association and dissociation steps were divided by the red dotted line. Real sensorgrams were in blue, green, orange, or purple. The fitting curves used for affinity calculation were in the red. (**M**) Number 9 hybridoma monoclonal antibody (K_D_ = 16 nM). (**N**) Number 42 hybridoma monoclonal antibody (K_D_ = 34.4 nM). (**O**) Number 49 hybridoma monoclonal antibody (K_D_ = 136 nM). (**P**) Number 81 hybridoma monoclonal antibody (K_D_ = 51.6 nM). (**Q**) Number 115 hybridoma monoclonal antibody (K_D_ = 45.3 nM). (**R**) Number 118 hybridoma monoclonal antibody (K_D_ = 250 nM). The three arrows in the figure are used to indicate the consecutive process in the antibody screening process.

After the booster immunization, Rabbit B showed a higher titer, species reactivity, and tissue specificity that was selected as the spleen donor to create hybridoma cells that generated a monoclonal antibody against Mb. Preliminary screening of hybridoma cells with the ability to specifically secrete anti-Mb to bind to His-Mb protein using peptide-based indirect ELISA. Then, WB testing was performed between His-Mb immunogen protein and 82 hybridoma supernatants that tested positive in ELISA. The results showed that the positive cell clone numbers were 36 (Figure 3A–D). The 36 positive hybridoma supernatants were incubated with mouse myocardium protein (Figure 3E,F) and human skeletal muscle protein (Figure 3G,H) to further confirm the tissue reactivity. Meanwhile, we also used rat spleen tissue protein to exclude non-specific binding, and the number 18 hybridoma cell was discarded (Figure S2A,B). Then, 29 clones were randomly selected from the remaining hybridoma cells to do affinity testing with rat skeletal muscle protein by immunoprecipitation experiment (Figure 3I–L). The IP results showed that the hybridoma supernatants of numbers 9, 42, 49, 81, 115, and 118 with a good enrichment effect needed to be further tested through the Fortebio biomolecular interaction detection system. An anti-Mb monoclonal antibody from number 9, 42, and 115 hybridoma supernatants bound more immunogen His-Mb protein and had a higher binding affinity (K_D_ = 16 nM, 34.4 nM, 45.3 nM, respectively), fast association rate (Kon = 2.52 × 10^4^ M^−1^ s^−1^, 2.84 × 10^4^ M^−1^ s^−1^, 2.78 × 10^4^ M^−1^ s^−1^, respectively), and slow dissociation rate (Kdis = 4.04 × 10^−4^ s^−1^, 9.75 × 10^−4^ s^−1^, 1.26 × 10^−3^ s^−1^, respectively) (Figure 3M,N,Q, Table S1). Monoclonal antibodies from numbers 49, 81, and 118 hybridoma supernatants bound more His-Mb protein, but also more dissociation (Figure 3O,P,R). In particular, a monoclonal antibody from number 49 and 118 hybridoma supernatants dissociated completely (Figure 3O,R). Consequently, number 115 hybridoma cells were obtained that produce the desired rabbit anti-Mb monoclonal antibody.

### 2.3. Preparation of Recombinant Rabbit Anti-Mb Monoclonal Antibody

**Figure 4:**
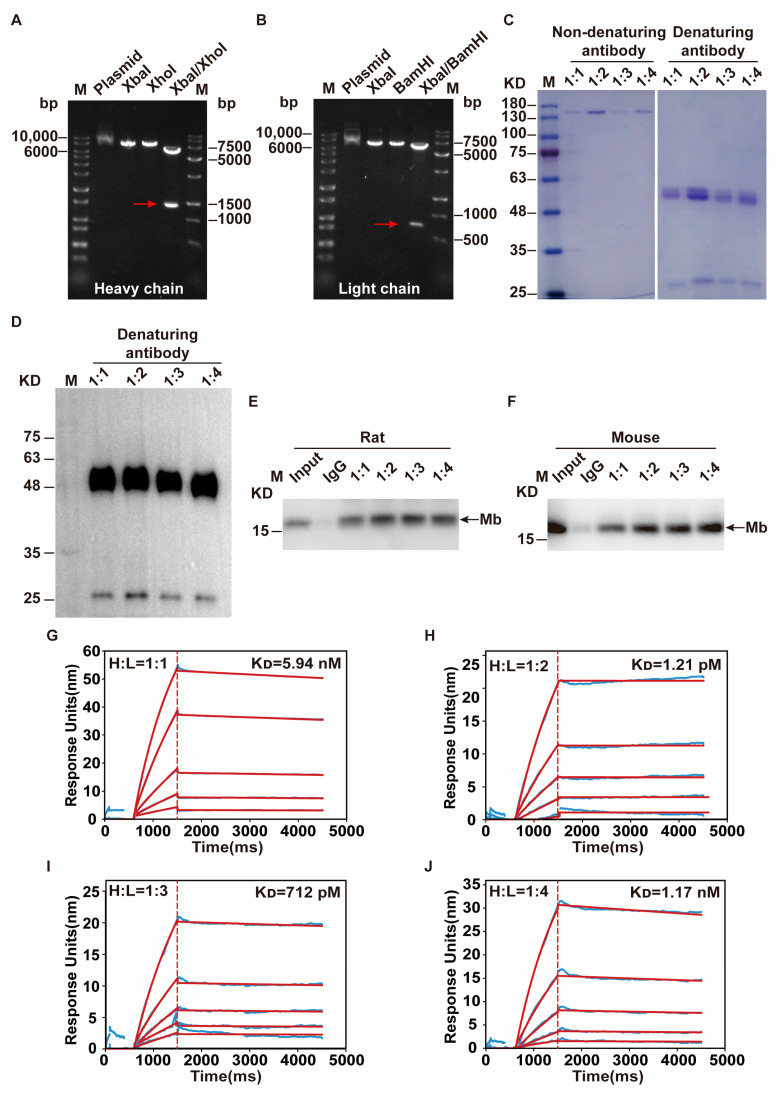
Preparation of recombinant rabbit anti-Mb monoclonal antibody. (**A**) Construction of heavy chain expression plasmid of recombinant anti-Mb monoclonal antibody. Xbal/XhoI double enzyme digestion of plasmid obtained target heavy chain gene. Red arrows indicate target bands. (**B**) Construction of light chain expression plasmid of recombinant anti-Mb monoclonal antibody. Xbal/BamHI double enzyme digestion of plasmid obtained target light chain gene. (**C**) The monoclonal antibodies from different transfection mass ratios of heavy chain and light chain were prepared under non-denaturing and denaturing conditions, separated by PAGE, and stained with Coomassie brilliant blue. Under the non-denaturing conditions, disulfide bridges stay intact, and the assembled monoclonal antibody that consisting of two heavy and two light chains with a size of about 150 kDa. (**D**) Using HRP-coupled goat anti-rabbit IgG secondary antibody (l:5000, ZSGB-BIO, Beijing, China, #ZB-5301) detected the heavy and light chains of the recombinant anti-Mb monoclonal antibody by WB assays that were separated and appear as obvious bands of 25 kDa and 50 kDa. (**E**,**F**) Immunoprecipitation of recombinant anti-Mb antibodies from different transfection mass ratios of heavy chain and light chain with rat or mouse skeletal muscle lysates. IgG antibody acted as a negative control. (**G**–**J**) SPR sensorgrams of recombinant anti-Mb antibodies from different transfection mass ratios of heavy chain and light chain (1:1, 1:2, 1:3, 1:4) to the His-Mb protein immobilized sensor chip. The raw data is shown as a light blue line, the calculated fit is shown as a red line.

We obtained the monoclonal antibody heavy and light chains gene sequence from DNA sequencing on number 115 hybridoma cells and then inserted them into the modified pKK44 vector via Xbal/XhoI and XbaI/BamHI restriction site, respectively. Heavy chain and light chain recombinant clones were confirmed by restriction enzyme digestion analyses in 0.8% agarose gel. After double enzyme digestion, the single and clear band could be seen at the target location. The molecular weight is about 1401 bp and 747 bp, respectively (Figure 4A,B). DNA sequencing results confirmed that the sequences were consistent with the gene in a heavy and light chain.

The function of the recombinant rabbit anti-Mb monoclonal antibody was associated with the specific pair of heavy and light chains. Therefore, we optimized the transfection ratio of heavy chain and light chain plasmid to transient expression in HEK-293F cells. In the gene shuffling testing, the transfection mass ratio of heavy and light chains chose 1:1, 1:2, 1:3, and 1:4, respectively, based on our previous experience. With non-denaturing PAGE, we could detect native IgG antibody bands containing two heavy and two light chains of approximately 150 kDa (Figure 4C, left). Meanwhile, we could detect a light chain of 25 kDa and a heavy chain of 50 kDa that was judged by Coomassie staining through the denaturing PAGE (Figure 4C, right). WB experiment showed the same results with the denaturing PAGE (Figure 4D). The immunoprecipitation results showed that recombinant rabbit anti-Mb monoclonal antibodies of different ratios all had the enrichment effect of Mb protein from the rat or mouse skeletal muscle lysates (Figure 4E,F). In addition, we utilized surface plasmon resonance (SPR) for the sensitive comparison of different transfection proportions on antibody binding affinities and kinetics to immunogen His-Mb protein. Recombinant rabbit anti-Mb monoclonal antibodies of different groups were found to readily associate with the His-Mb protein and a slow dissociation phase was observed, showing a relatively stable interaction. The equilibrium dissociation constant (K_D_) calculated from a steady state analysis were 5.94 nM, 1.21 pM, 712 pM, and 1.17 nM in 1:1, 1:2, 1:3, and 1:4 of heavy chain to light chain, respectively (Figure 4G–J, Table S2). Meanwhile, the transfection mass ratio of heavy and light chains about 1:2 had the highest affinity (K_D_ = 1.21 pM) (Figure 4H). Many commercial antibodies are difficult to achieve such high affinity. The K_D_ value of the anti-Myoglobin antibody (Abcam, Cat: ab77232) is 178 pM, the K_D_ value of anti-TGF-β (Abcam, Cat: ab124894) is 86.4 pM, the K_D_ value of anti-cytokeratin 15 antibodies (Abcam, Cat: ab52816) is 560 pM. In addition, the K_D_ value of recombinant rabbit anti-Mb monoclonal antibody with recombinant human Mb and native horse Mb were 6.40 μM and 87.33 μM, respectively (Figure S3A,B). The yields were about 40 mg/L. Therefore, we chose the transfection mass ratio of heavy and light chain about 1:2 to generate recombinant rabbit anti-Mb monoclonal antibody.

**Figure 5:**
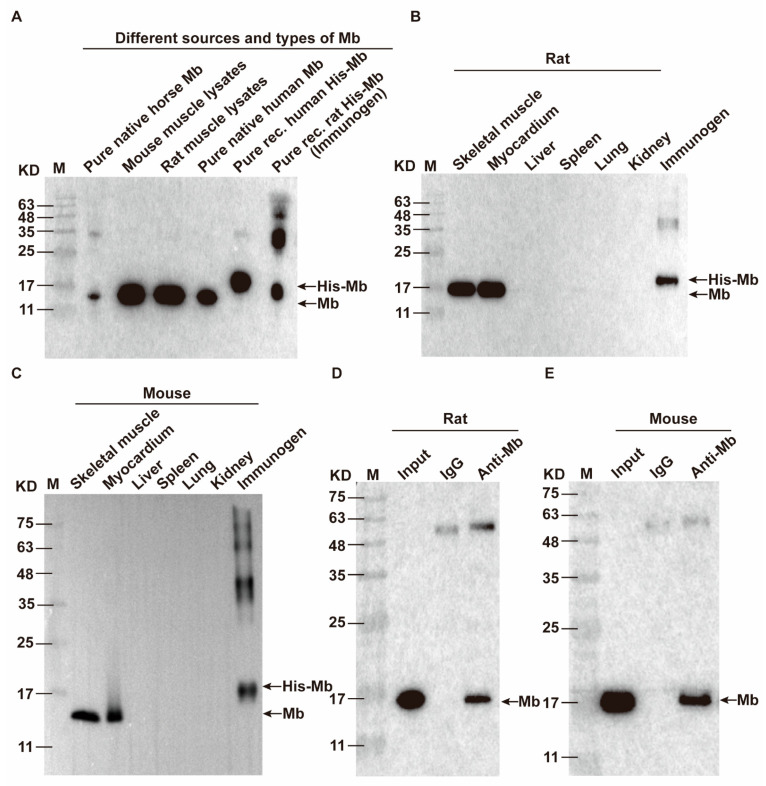
Validation of the species reactivity and tissue specificity of recombinant rabbit anti-Mb monoclonal antibody of transfection mass ratio of heavy chain and light chain is 1:2. (**A**) WB analysis for species reactivity of recombinant rabbit anti-Mb monoclonal antibody (transfection mass ratio of heavy chain and light chain = 1:2). Lane 1: M is protein marker (11–245 kDa); Lane 2: Pure native horse Mb (Beijing Solarbio Science & Technology Co., Ltd., #M8090); Lane 3: Mouse muscle lysates (skeletal muscle lysis mixture of mice); Lane 4: Rat muscle lysates (skeletal muscle lysis mixture of rats); Lane 5: Pure native human Mb (Abcam, #ab96036); Lane 6: Pure recombinant human His-Mb (OkayBio, #K2916); Lane 7: Pure recombinant rat His-Mb protein (immunogen). The protein amounts of pure native horse Mb, mouse skeletal muscle lysate (mixture), rat skeletal muscle lysate (mixture), pure native human Mb, pure recombinant human Mb, and immunogen (rat His-Mb) are 5 µg (pure protein), 50 µg (total protein), 50 µg (total protein), 0.66 µg (pure protein), 0.264 µg (pure protein), and 0.21 µg (pure protein), respectively. (**B**,**C**) The Mb protein expression in skeletal muscle, myocardium, liver, spleen, lung, and kidney of normal rats or mice was detected by WB using recombinant rabbit anti-Mb monoclonal antibody. Immunogen His-Mb protein acted as a positive control. (**D**,**E**) Immunoprecipitation of the recombinant rabbit anti-Mb monoclonal antibody with rat or mouse skeletal muscle lysates. IgG antibody acted as a negative control.

Then we detected the species reactivity and tissue specificity of the recombinant rabbit anti-Mb monoclonal antibody. This recombinant monoclonal antibody had broad species reactivity with horses, mice, rats, and humans (Figure 5A). Meanwhile, the recombinant monoclonal antibody had good tissue specificity that only existed in the skeletal muscle and myocardium, but not in the liver, spleen, lung, and kidney of normal mice or rats (Figure 5B,C). In addition, this monoclonal antibody also performed well in the immunoprecipitation experiment with rat or mouse skeletal muscle lysates (Figure 5D,E). Overall, we obtained recombinant rabbit anti-Mb monoclonal antibodies with high affinity, broad species reactivity, tissue specificity, and sensitivity.

### 2.4. Functional Validation of Recombinant Rabbit Anti-Mb Monoclonal Antibody in RM-AKI

**Figure 6:**
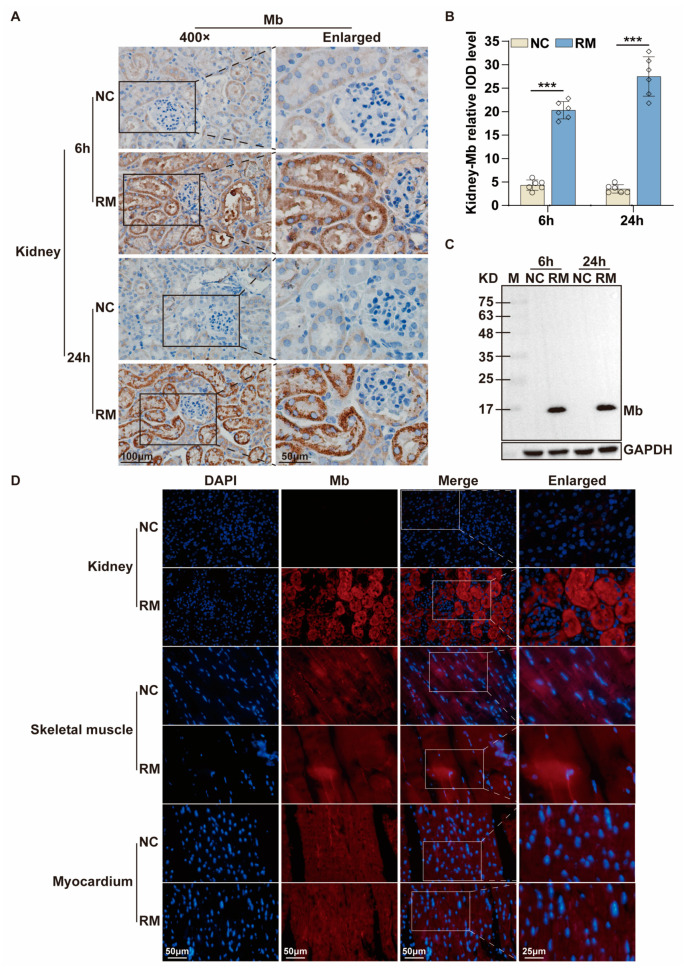
Application of recombinant rabbit anti-Mb monoclonal antibody in RM-AKI. (**A**) Application of the immunohistochemistry staining detected the Mb protein distribution in the kidney tissue sections of RM-AKI mice with recombinant rabbit anti-Mb monoclonal antibody (×400). (**B**) The integrated optical density (IOD) of Mb protein in each group (*n* = 6; ***, *p* < 0.001). (**C**) WB assays detected the changes in Mb protein expression in the kidney of RM-AKI mice with recombinant rabbit anti-Mb monoclonal antibody. (**D**) Application of the immunofluorescence staining detected the Mb protein distribution in the kidney, skeletal muscle, and myocardium tissue sections of RM-AKI mice with recombinant rabbit anti-Mb monoclonal antibody.

To further verified that application effect of the prepared recombinant rabbit anti-Mb monoclonal antibody in the kidney, skeletal muscle, and myocardium of mice RM-AKI disease. Immunohistochemistry staining results showed the location information of the Mb protein that was deposited in the renal tubules of RM-AKI (Figure 6A). Meanwhile, the staining intensity of this antibody could reflect the aggravation of the disease process. The deposition of Mb protein in the renal tubules in 24 h robust exceeds 6 h of RM-AKI (Figure 6A,B). Then WB results showed that Mb protein accumulated in the kidney of mouse RM-AKI and the bands of the same size as the target protein (Figure 6C). At the same time, there was no specific background signal in the negative control group (Figure 6A,C). In addition, immunofluorescence staining analysis obtained the same results. The average fluorescence intensity of Mb protein in the kidney of the mouse RM-AKI group was significantly higher than that in the control group (Figure 6D, upper). Moreover, Immunofluorescence analysis also showed that the fluorescence signal of Mb protein detected in the skeletal muscle and myocardium of the RM-AKI group was not significantly different from the control group. However, from the morphological point of view, the skeletal muscle fibers of the control group were evenly arranged and dense. In contrast, the muscle fibers of the RM-AKI group were uneven in size and showed severe myofilament rupture (Figure 6D, middle and lower). In other words, this recombinant monoclonal antibody could be used to detect the Mb protein in RM-AKI disease.

In short, our study showed that recombinant rabbit anti-Mb monoclonal antibody could locate the Mb protein in the key kidney tissue of RM-AKI disease, indicating that this recombinant monoclonal antibody had the advantage of high specificity and more applications.

## 3. Discussion

In this study, we successfully obtained high purity and enough amount of immunogen His-Mb fusion protein. Furthermore, high quality and titer anti-Mb polyclonal antibodies were generated in immunized rabbits. Through the hybridoma technique, after a series of specificity and affinity screening, one number 115 hybridoma cell with a K_D_ value of 45.3 nM was obtained. With the help of DNA sequencing and a molecular clonal, monoclonal antibody heavy and light chains plasmid was constructed. At last, we obtained recombinant rabbit anti-rat Mb monoclonal antibody with high affinity, broad species reactivity, tissue specificity, sensitivity, and multifaceted application. This antibody had a good performance in the detection of Mb protein deposition of RM-AKI disease.

Recombinant proteins have important commercial value in diagnosis and therapeutic use [25]. There are many different protein expression systems, which can be broadly divided into prokaryotic and eukaryotic expression systems. *Escherichia coli* (*E. coli*) is one of the most choices for recombinant protein expression in prokaryotic. It has the advantage of simple operation, background clear, high expression, and cost-effectively. However, the corresponding disadvantages are that expressed proteins cannot secrete outside the cell, with endotoxin, and lack post-translational modifications [26,27,28]. In contrast, the eukaryotic expression system compensates for the shortcomings of the prokaryotic expression system. Eukaryotes offer diverse organisms ranging from yeast, plants, animals, or human cells to express recombinant protein [29]. Commonly adopted eukaryotic expression systems are based on stably transfected adherent CHO cells or transient expression in mammalian nonadherent cell lines [30]. In our study, transient expression in human HEK-293F cells was chosen to express and purify the immunogen His-Mb fusion protein. Obtaining stable expressed recombinant His-Mb proteins is the first step for animal immunization. Additionally, high-quality His-Mb protein can provide ample support for the generation of polyclonal antibodies. The eukaryotic HEK-293F cell expression system makes it easy to obtain sufficient amounts of highly active proteins in a near-native state. After purification through a nickel affinity chromatography column, we obtained the ideal purity (about 95%), concentration, and quantity of His-Mb protein. This is one of the pivotal prerequisites for the preparation of high- titer anti-Mb serum successfully [31].

Rabbits and mice are the most common hosts for generating antibodies. In general, compared to mice, rabbits produced superior antibodies for several reasons. First, rabbits have a high success rate with a larger range of immune response antigens such as small molecules and haptens that are often non-immunogenic in mice [32,33,34,35]. In other words, rabbit antiserum will contain a wider variety of antibodies. Second, rabbits have more nature antibody repertoire diversity and the spleen are larger than the mouse [17,18,36]. Antibodies that are produced in rabbits often recognize more epitopes per protein antigen than mouse antibodies. Compared to mice, due to the larger body size, more than 50 times spleen B cells can be recovered from rabbits [17]. Third, rabbit antibodies seem to have a better affinity and specificity for antigens than mouse antibodies [35]. Forth, for the application, some researchers consider that rabbit antibodies perform better effect in immunohistochemistry and immunocytochemistry assays [35]. Therefore, we immunized New Zealand white rabbits with purified His-Mb protein as an immunogen. We successfully obtained a rabbit anti-rat Mb polyclonal antibody with a titer of more than 1:256,000 that was tested by ELISA. The plasma-derived anti-Mb polyclonal antibodies showed a wide range of species cross-reactivity with horses, mice, rats, and humans. Meanwhile, it had high tissue specificity that could specifically target Mb-rich skeletal muscle and myocardial tissue, but not liver, spleen, lung, and kidney tissue without Mb protein expression in normal situations. Moreover, the plasma-derived anti-Mb polyclonal antibody had sensitivity in detecting myoglobin deposition in the kidney tissues of mice RM-AKI model by WB and immunohistochemistry assays.

Although plasma-derived polyclonal antibodies are still a preferred choice to treat many selected clinical diseases. However, plasma-derived polyclonal antibodies have many limitations including safety issues, batch-to-batch variation, lack of specificity, the rarity of suitable plasma, cost, and limited clinical applications [37]. For a number of years, the life science industry has been in the grips of a reproducibility crisis. Meanwhile, from a pharmaceutical point of view, there is a need for molecularly defined and reproducible antibody products. The advent of hybridoma techniques, as a crucial milestone, revolutionized the antibody field. This method allows the production of purity and highly specific monoclonal antibody in vitro [38]. Compared with plasma-derived polyclonal antibodies, monoclonal antibodies are considered more specific [32,39]. Moreover, monoclonal antibody provides some additional advantages of high binding affinity and safety [38]. Although the polyclonal antibody also has a good effect on AKI detection in this study, we still need to prepare monoclonal antibodies considering their various limitations, late commercialization, and safety. Therefore, in our study, after screening by ELISA and WB assays, we obtained 36 positive hybridoma cells that could produce rabbit anti-Mb monoclonal antibodies. Then, these hybridoma supernatants interact with the myocardium, skeletal muscle, and spleen to perform tissue cross-reactivity testing. All the monoclonal antibody from clones has a good reactivity with mouse myocardium tissues. However, for some clones, the species’ reactivity is limited. It could not react with the human skeletal muscle. This, again, verified the diverse and heterogeneity of plasma-derived polyclonal antibodies [40]. Meanwhile, 29 hybridoma supernatants that have positive reactivity with mouse myocardium tissue lysates were randomly selected to do the binding affinity testing with rat skeletal muscle lysates by immunoprecipitation experiments. Number 9, 42, 49, 81, 115, and 118 hybridoma supernatants with good reactivity and affinity were selected to do the more accurate binding affinity testing by Fortebio assays. At last, number 115 hybridoma cells were obtained that produce the desired anit-Mb monoclonal antibody with a high binding affinity (K_D_ = 45.3 nM), and slow association rate (Kdis = 1.26 × 10^−3^ s^−1^).

Everything has two sides. Although monoclonal antibody has enormous commercial value in the diagnosis and treatment of the disease with a simple antigen. However, due to their monospecific nature, the monoclonal antibody lacks efficacy in neutralizing, killing, or eliminating complex target antigens-associated diseases, such as cancer cells, a virus with multiple strains, and infectious disease agents [41,42,43,44]. Therefore, the best antibody for the research would depend on time, budget, and application. For this study, the goal is to rapidly obtain a large quantity of reproducible monoclonal antibodies with high specificity and high affinity to be used in Mb-associated disease (for example RM-AKI) diagnosis. So, based on the above positive results, we hope to move forward with this antibody and get recombinant monoclonal antibodies.

Compared with plasma-derived polyclonal antibodies and hybridoma-derived monoclonal antibodies, recombinant antibodies may provide several advantages. The supply limitation of plasma-derived polyclonal antibodies is batch-to-batch variation, and the production limitation of hybridoma-derived monoclonal antibodies is technically demanding and not straightforward enough sometimes [45]. The advantages of the recombinant antibody include the speed of antibody generation, altering affinity and specificity’s possibility, the ability to generate novel and rare functionalities, humanization, and so on [46]. Therefore, due to the lower cost for long-term production and safe expression systems, a recombinant monoclonal antibody is a very promising technology to complement or replace hybridoma technology.

After single-cell sequencing, monoclonal antibody heavy and light chain gene sequences were obtained and cloned into the modified pKK44 expression vector, respectively. Compared with phage-display-based approaches, the original cognate pairing of the heavy and light chain of the antibody is preserved and has high affinity [37]. In addition, for high biological activity, the recombinant monoclonal antibody should be folded, assembled, and appropriately glycosylated [47]. Therefore, in order to obtain high-quality recombinant monoclonal antibodies, both heavy and light chain genes need to be transcribed simultaneously after co-transfection, and proportionally coordinated. This is the key challenge of successfully producing recombinant monoclonal antibodies from transfected HEK-293F cells. So, the transfection ratio of a heavy and light chain is crucial. After optimization, the mass ratio of 1:2 on heavy and light chain expression plasmid that co-transfected into HEK-293F cells were selected. Through the binding affinity test by SPR experiment, this recombinant anti-Mb monoclonal antibody has a 10,000-fold improvement (K_D_ = 1.21 × 10^−12^ M) in immunogen His-Mb protein binding affinity with respect to a monoclonal antibody of hybridoma supernatants (K_D_ = 4.53 × 10^−8^ M). Meanwhile, this recombinant rabbit anti-Mb monoclonal antibody still had good tissue specificity and broad species reactivity with horses, mice, rats, and humans. Moreover, this anti-Mb antibody has good applications in immunoprecipitation experiments with rat or mouse skeletal muscle lysates. Overall, we obtained recombinant rabbit anti-Mb monoclonal antibodies with high affinity, broad species reactivity, tissue specificity, and sensitivity.

As we all know, Mb is the main constituent of skeletal muscle and is a vital factor in the pathophysiology of rhabdomyolysis [48]. Meanwhile, it is contributing to renal damage in rhabdomyolysis [49]. Previous research indicated that Mb causes RM-AKI through three mechanisms including Mb toxicity, iron dissociation and free radicals release, and lipid peroxidation [10]. So, the detection of Mb protein deposition is a key factor for the basic research of Mb-associated diseases, such as RM-AKI or CS-AKI [50]. In addition, Mb is the key earliest biomarker for ischemic heart disease [12,51] and the routine biochemical indicator for hospital testing of rhabdomyolysis patients. However, the current commercial recombinant rabbit anit-Mb monoclonal antibody is difficult to meet the requirements of high affinity, broad species reactivity, and diverse application at the same time. The immunogen is truncated human Mb (P02144), not Rat Mb (Q9QZ76). We generate the recombinant rabbit anit-rat Mb monoclonal antibody not only has extremely high affinity (K_D_ = 1.21 pM) but also has broad species reactivity (horse, mice, rat, and human). Meanwhile, this anti-Mb monoclonal antibody was characterized and tested in different applications such as WB, immunoprecipitation, immunochemistry, as well as immunofluorescence confirming its high quality. It may be used detect Mb protein deposition in the kidney of RM-AKI mice, as well as increased Mb protein expression in the blood, compared with normal mice. Due to the limitation of clinical AKI samples, we did not carry out relevant tests, but our results show that the antibody has good species reactivity with humans. Therefore, this anti-Mb monoclonal antibody has a very strong role in tracer myoglobin. If it is made into a biochemical test kit, it can be used for rapid diagnosis of clinical RM-AKI-related diseases. Degradation reaction may cause heterogeneous recombinant monoclonal antibodies [52]. So, subsequent detection and improvement of antibody stability are required. Meanwhile, due to the extremely high binding affinity of this anti-Mb antibody, it may be used as a neutralizing antibody for disease treatment. To enable successful therapeutics, this anti-Mb monoclonal antibody should need to be humanized [53]. This is what we need to continue to push forward in the future.

## 4. Materials and Methods

### 4.1. Experimental Animals and Cells

New Zealand white rabbits (6 months old) and C57BL/6 J mice (about 20 g, 8–10 weeks old) were housed in a pathogen-free environment with a 12 h light/dark cycle and free access to food and water. Rabbits were used for immunization experiments, and mice were used to establish rhabdomyolysis associated acute kidney injury (RM-AKI) model. All mice were randomly divided into different groups. For RM-AKI group mice, 50% glycerin solution (prepared with normal saline) was injected into the femoral muscles of both hind limbs at half a dose (8 mL/kg). The mice in the Control group (NC) were intramuscularly injected with saline (8 mL/kg) [54]. After 6 h and 24 h of injection, the mice were anesthetized, and their blood and kidney tissues were collected and stored at −80 °C for subsequent experiments.

Human embryonic kidney 293F (HEK-293F) cells (ATCC) were cultured at 37 °C in humidified air with 5% CO_2_. Authentication was confirmed by the suppliers. The cell morphology was examined by a light microscope, and the mycoplasma was confirmed as negative.

### 4.2. Multiple Sequence Alignment Analysis of Mb Protein

The amino acid sequences of Mb protein in common species (*Rattus*, *Mus*, *Homo*, *Equus*, *Sus*, *Bos*, *Ovis*, *Canis*, *Oryctolagus*, *Macaca*, *Gallus*, and *Danio*) were downloaded from the National Center for Biotechnology Information Search database (NCBI). The identity and similarity analysis among these twelve amino acid sequences was compared by ClustalX and DNAMAN.

### 4.3. Expression and Purification of Immunogen His-Mb Protein

The CDS region sequence (465 bp) of the rat (*Rattus norvegicus*) myoglobin gene sequence (Gene ID: 59108) was downloaded from the GeneBank database. EcoRI enzyme digestion site and protective bases were added in the front of the initial codon ATG. 6× His sequences were added in the front of the termination codon TGA. BamHI enzyme digestion site and protective bases were added behind the termination codon to synthesize gene fragments encoding rat myoglobin (Sangon Biotech Co., Ltd., Shanghai, China). Then the gene fragment was inserted into the pTT5 vector (Ke Lei Biological Technology Co., Ltd., Shanghai, China, #kl-zl-0637) to obtain the expression plasmid pTT5-Mb. QuickCutTM EcoRI (Takara, Obe, Japan, #1611) and QuickCutTM BamHI (Takara, #1605) were subjected to double enzyme digestion analysis for initial identification and further sequenced by Sangon Biotech Co., Ltd. After the sequencing was confirmed, the pTT5-Mb plasmid was transfected into HEK-293F cells using LipofectamineTM 2000 (ThermoFisher, Waltham, MA, USA, #11668019) according to manufacturer’s instructions. The cell culture medium supernatant was collected after transfection for 4872 h. Then, the supernatant was purified with Ni Sepharose High Performance (Cytiva, Marlborough, MA, USA #17526801) and concentrated using an ultrafiltration centrifugal tube (Millipore, Burlington, MA, USA, #UFC900396) to obtain the purified His-Mb protein.

### 4.4. Preparation of Anti-Mb Polyclonal Antibody

Three healthy New Zealand white rabbits were immunized and numbered Rabbit A, Rabbit B, and Rabbit C [55]. Ear vein blood was drawn before immunization as a negative control for subsequent serum tests. The purified Mb protein (0.8 mg/mL) was used as an immunogen and mixed with an equal volume of complete Freund’s adjuvant (SIGMA, St. Louis, MO, USA, #F5881). After full emulsification, each rabbit was injected subcutaneously with 1 mL at the back for initial immunization. After 3 weeks, the immunogen protein (0.4 mg/mL) was fully emulsified with the same volume of incomplete Freund’s adjuvant (SIGMA, #F5506), and each rabbit was injected with 1 mL for enhanced immunity. Thereafter, booster immunization was carried out every 2 weeks, 4 times in total. At the last booster immunization, 1 mL of immunogen protein (0.4 mg/mL) was injected into the ear vein without adding an adjuvant. After 4 days, blood was taken from the carotid, and serum was collected to detect the antibody titer. Then the rabbit spleen was taken out and stored in a centrifuge tube containing 10 mL RPMI 1640 (Shandong Sparkjade Biotechnology Co., Ltd., Qingdao, China, #CF0002-500 ML) culture medium, and quickly sent to the cell culture room for the next treatment.

### 4.5. Determination of the Titer of Anti-Mb Polyclonal Antibody by ELISA

1 μg/mL His-Mb immunogen protein was coated in the 96-well enzyme label plate (NEST Biotechnology Co., Ltd., Wuxi, China) at 4 °C overnight. Then 300 μL 1% BSA (Beijing Solarbio Science & Technology Co., Ltd., Beijing, China #A8010) sealing solution was added and incubated at 37 °C for 1 h. After that, added 200 μL test serum with a dilution ratio of 1:250, 1:1000, 1: 4000, 1:16,000, 1:64,000, and 1:256,000. The serum before immunization was used as the negative control and incubated at 37 °C for 1 h. 100 μL HRP-coupled secondary antibody (l:8000, ZSGB-BIO, #ZB-5301) was added and incubated at 37 °C for 1 h. Finally, added 100 μL TMB substrate and incubated at 37 °C in a dark place for 10–20 min, and then added 50 μL HCL to stop the reaction. The absorbance value of each hole at the wavelength of 450 nm (OD450) was detected by an enzyme labeling instrument (Hangzhou Allsheng Instruments Co., Ltd., Hangzhou, China, FlexA-200).

### 4.6. Screening of Hybridoma-Derived Rabbit Anti-Mb Monoclonal Antibody

After antibody titer and specificity detection, the spleen of Rabbit B was selected and prepared into a single-cell suspension. The rabbit active splenocytes were isolated and electrofused with rabbit myeloma cells to develop hybridoma cells, which were cultured overnight in RPMI 1640 medium. Then the cells were cultured for 10–14 days at 37 °C and 5% CO_2_ in a medium that was uniformly mixed with selective medium HAT (ThermoFisher, #21060017) and semi-solid medium. Single clones were selected and placed into 96-well plates under the microscope, and then 200 μL selective medium HAT was added for further culture. When the clone grew to about 80%, the antibody titer in the supernatant was detected by indirect ELISA and 30–50 monoclonal hybridoma cell lines were screened for liquid expansion culture. The positive hybridoma cells supernatant was further screened by WB, IP, and Fortebio detection. Finally, an optimal hybridoma cell line was selected.

### 4.7. Expression and Purification of Recombinant Anti-Mb Monoclonal Antibody

Single-cell sequencing was performed on the final selected hybridoma cell to obtain the heavy chain and light chain gene sequences of the antibody. TurboCapture 96 mRNA Kit (Qiagen, Hilden, Germany, #72251) was used to extract the mRNA of hybridoma cell according to the manufacturer’s instructions, and the heavy chain and light chain gene fragments of Mb monoclonal antibody were obtained by PCR amplification, respectively. The antibody gene fragments were inserted into the modified pKK44 vector by homologous recombination to construct the expression plasmids of the monoclonal antibody heavy chain and light chain. Then Lipofectamine™ 2000 transfection reagent was used to mix the heavy chain and light chain expression plasmids in a mass ratio of 1:1, 1:2, 1:3, and 1:4, respectively, and transfected into HEK-293F cells. After 4 days, the supernatant was collected by centrifugation and purified by Protein A affinity column (GE Healthcare). Finally, a high-purity recombinant anti-Mb monoclonal antibody was obtained.

### 4.8. PCR and Quantitative Real-Time PCR (qPCR)

The total RNA of kidney tissue was extracted by TRIeasy™ LS Total RNA Extraction Reagent (YEASEN, Shanghai, China, #19201ES60), and the mRNA of hybridoma cells was extracted by TurboCapture 96 mRNA Kit (Qiagen, #72251) according to the manufacturer’s instructions. The purity and concentration of the extracted RNA samples were detected by Nanodrop One. Reverse transcription into cDNA was performed using the Hifair^®^ II 1st Strand cDNA Synthesis Kit (YEASEN, #11141ES60). Then the 2 × Hieff^®^ PCR Master Mix (With Dye) kit (YEASEN, #10102ES08) was used to perform PCR with Eppendorf Mastercycle nexus GX2 PCR. The Cycle-pure kit (Omega, Norcross, GA, USA, #D6492-01) was used to purify PCR products. A Hieff^®^ qPCR SYBR Green Master Mix (No Rox) (YEASEN, #11201ES03) kit was used to perform qPCR with LightCycler^®^ 96 instrument (Roche, Basel, Switzerland). The 2^−ΔΔCt^ method was applied to calculate relative expression levels. Primer information is shown in Table S3.

### 4.9. Western Blotting (WB)

Horse myoglobin is a commercial pure protein (Beijing Solarbio Science & Technology Co., Ltd., #M8090), mouse and rat myoglobin come from skeletal muscle lysis mixture of mice and rats, native human myoglobin is a commercial pure protein (Abcam, Cambridge, UK, #ab96036), and recombinant human myoglobin is a commercial pure protein (OkayBio, Nanjing, China, #K2916). The protein amounts of pure native horse Mb, mouse skeletal muscle lysate (mixture), rat skeletal muscle lysate (mixture), pure native human Mb, pure recombinant human Mb, and immunogen (rat His-Mb) are 5 µg (pure protein), 50 µg (total protein), 50 µg (total protein), 0.66 µg (pure protein), 0.264 µg (pure protein), and 0.21 µg (pure protein), respectively. The protein was separated by SDS-PAGE and subsequently transferred onto the PVDF membrane. Then, the PVDF membrane was sealed in 5% skimmed milk solution (Shandong Sparkjade Biotechnology Co., Ltd., #ED0019) for 2 h at room temperature, and incubated with the anti-Mb antibody (1:5000) and anti-GAPDH antibody (1:5000, Beijing Solarbio Science & Technology Co., Ltd., #K200103M) overnight on a shaker at 4 °C. The membrane was incubated with the secondary antibody (1:5000, Sunkine Biotech, Shanghai, China, #LK2001, #LK2003) labeled with HRP at room temperature for 1 h. Protein bands were stained with the highly sensitive ECL chemiluminescence detection kit (YEASEN, #3622ES60). Finally, Tanon 5200 Multiple Detection System was used for imaging. The intensity value of bands was analyzed by the Tanon Gel-Pro Analyzer system.

### 4.10. Immunoprecipitation (IP)

IP for His-Mb: Purified His-Mb protein was incubated with protein A magnetic beads and mouse anti-6 × His tag monoclonal antibody (Abcam, #ab18184)/mouse anti-IgG antibody (Abcam, #ab190475) overnight with shaking at 4 °C. The magnet was used to adsorb the beads, and the sample was washed three times with a binding buffer. Then, WB analysis was performed using rabbit anti-Mb monoclonal antibodies (1:2000, Abcam, #ab77232) and HRP-coupled goat anti-rabbit IgG secondary antibody (l:5000, ZSGB-BIO, #ZB-5301).

IP for Anti-Mb antibody: Total proteins from rat and mouse muscle tissue were extracted with IP lysis buffer and incubated with protein A magnetic beads and rabbit anti-Mb polyclonal/monoclonal antibody/rabbit anti-IgG antibody (Abcam, #ab172730) overnight with shaking at 4 °C. The magnet was used to adsorb the beads, and the sample was washed three times in a binding buffer. Moreover, WB analysis was performed using mouse anti-Mb antibody (1:100, Santa Cruz, Dallas, TX, USA, #sc-74525).

### 4.11. Hematoxylin-Eosin (HE)

The isolated kidney tissue samples were fixed in 4% paraformaldehyde (Beijing Solarbio Science & Technology Co., Ltd., #P1110). Then the tissue was dehydrated and embedded in paraffin, and sectioned into 4 μm thickness. After staining with Hematoxylin-Eosin (HE) Stain Kit (Beijing Solarbio Science & Technology Co., Ltd., #G1120) and dehydration, the pathological changes of renal tissue were observed and evaluated using light microscopy.

### 4.12. Immunohistochemistry and Immunofluorescence

The tissue sections of the RM-AKI mice were deparaffinized with xylene, rehydrated with alcohol, incubated with 3% hydrogen peroxide, and heated for antigen repair. Plasma membranes were permeabilized with 0.5% Triton X-100 (Beijing Solarbio Science & Technology Co., Ltd., #T8200). Nonspecific binding sites were blocked using 5% goat serum (Beijing Solarbio Science & Technology Co., Ltd., #SL038). Sections were incubated with anti-Mb polyclonal/monoclonal antibody (1:500) at 4 °C overnight. For immunohistochemistry, the sections were incubated with HRP-coupled secondary antibody (l:200, ZSGB-BIO, #ZB-5301) at room temperature for 1 h. After washing, the sections were stained with DAB (Beijing Solarbio Science & Technology Co., Ltd., #DA1015), and the nuclei were re-stained with hematoxylin. After mounting with neutral gum, the sections were observed and photographed. For immunofluorescence, the sections were incubated with secondary antibodies coupled to Alexa Fluor 488/594 (1:200, ZSGB-BIO, #ZF-0516) at room temperature for 1 h. The cell nucleus was stained by 4′,6-diamidino-2-phenylindole (DAPI) (Beijing Solarbio Science & Technology Co., Ltd., #C0060) in the dark. At last, the sections were observed and photographed by confocal microscopy (Nikon Corporation, Tokyo, Japan, A1).

### 4.13. Biochemistry Analysis

Centrifuge mouse blood samples at 3000 rpm for 15 min to collect serum. Use an automated biochemical analyzer (iMagic V7, Icubio) to detect the serum levels of creatine kinase (CK), Myoglobin (Mb), blood urea nitrogen (BUN), and serum creatinine (Scr) by commercial assay kits from Shenzhen Derui Biotechnology Co., Ltd. (Shenzhen, China) following manufacturer′s protocols.

### 4.14. Surface Plasmon Resonance (SPR)

The affinity between His-Mb protein and anti-Mb antibodies from different transfection ratios of heavy and light chain plasmid were measured at room temperature using a Biacore T200 SPR instrument. All proteins used for kinetic analysis were exchanged to the buffer of phosphate-buffered saline (pH 7.4). The Protein A chip (GE Healthcare) was coupled with different antibodies. Gradient concentrations of His-Mb protein (from 500 nM to 31.25 nM with 2-fold dilution) then flowed over the chip surface. After each cycle, the sensor surface was regenerated. The data was collected and the affinity was calculated.

### 4.15. Bio-Layer Interferometry (BLI) Binding Assay

To study the interactions between His-Mb protein and purified anti-Mb antibodies from different hybridoma supernatant, we used ForteBio Octet Red96e and CM5 sensors (ForteBio). The experiment was conducted at 25 °C in a phosphate-buffered saline buffer. Anti-Mb antibodies were immobilized on CM5 sensor tips according to the manufacturer’s recommendations. His-Mb protein serves as the mobile phase. Another reference sensor without immobilized protein was subjected to the identical procedure for double referencing and subtracting non-specific His-Mb protein binding with the sensor material. After a stable baseline was observed, association and dissociation were monitored. The obtained data were analyzed, and the K_D_ value for substrate binding was calculated.

### 4.16. Mass Spectrometry Analysis

Mix 1 µg antibody with 5 × SDS-PAGE Protein Loading Buffer (YEASEN, # 20315ES05) and boil. SDS-PAGE and Coomassie Brilliant Blue staining were performed. The light chain and heavy chain of the antibody were cut off, digested with trypsin, and sent to BGI company for mass spectrometry analysis by MicrOTOF-QII equipment (BrukerDaltonics, Billerica, MA, USA).

### 4.17. Statistical Analysis

Each experiment was repeated three times, and continuous variables with normal distribution were presented as mean ± standard deviation (SD). For two or more groups, two-way ANOVA followed by Tukey’s multiple comparisons test were used to identify the differences. Three levels of statistical significance (* *p* < 0.05; ** *p* < 0.01; *** *p* < 0.001; ns: no significance) were set. *p* < 0.05 was considered to be statistically significant. All data statistical analysis and image generation were performed in GraphPad Prism 8 software.

## 5. Conclusions

In summary, we successfully constructed the pTT5-Mb expression plasmid and expressed and purified the immunogen His-Mb fusion protein. The pure His-Mb protein could be used to prepare rabbit anti-rat Mb polyclonal antibodies of animal immunization techniques. The plasma-derived rabbit anti-Mb polyclonal antibodies had a high titer which was detected by ELISA. Meanwhile, rabbit anti-Mb polyclonal antibodies showed a broad species reactivity and high tissue specificity. Meanwhile, these polyclonal antibodies could be applied to detect the Mb protein expression level that increased significantly in the kidney of RM-AKI mice. Then after a series of specificity and affinity screening about hybridoma monoclonal antibody, we obtained one hybridoma cell whose number is 115 and the equilibrium dissociation constant (K_D_) is 4.53 × 10^−8^ M. Through DNA sequencing and a molecular clonal, monoclonal antibody heavy and light chain plasmids were constructed. The optimized mass ratio of heavy chain and light chain plasmid to transient expression in HEK-293F cells was 1:2. The recombinant rabbit anti-Mb monoclonal antibody affinity is extraordinarily high, and the K_D_ value is 1.21 × 10^–12^ M. Meanwhile, this recombinant rabbit anti-Mb monoclonal antibody had broad species reactivity (mouse, rat, human, and horse) and good tissues specificity (skeletal muscle and myocardium). For the application, WB, immunohistochemistry, and immunofluorescence staining methods were well used to detect the Mb protein level in the kidney, myocardium, and skeletal muscle of RM-AKI. Overall, we obtained a novel recombinant rabbit anti-rat Mb monoclonal antibody with high affinity, broad species reactivity, tissue specificity, sensitivity, and multifaceted application.

## Data Availability

All data that support the findings of this study are available from the corresponding authors upon reasonable request.

## Supplementary Materials

The following supporting information can be downloaded at: https://www.mdpi.com/article/10.3390/ijms24097822/s1.

## Abbreviations

Mb	myoglobin
RM	rhabdomyolysis
AKI	acute kidney injury
RM-AKI	rhabdomyolysis associated acute kidney injury
CS-AKI	crush syndrome associated acute kidney injury
BUN	blood urea nitrogen
Scr	serum creatinine
CK	creatine kinase
KIM-1	kidney injury molecule-1
NGAL	neutrophil gelatinase-associated lipocalin
WB	western blotting
HE	hematoxylin-eosin
IP	immunoprecipitation
SPR	surface plasmon resonance
