# Supplementary material for: A Novel Rabbit Anti-Myoglobin Monoclonal Antibody’s Potential Application in Rhabdomyolysis Associated Acute Kidney Injury
Source: Int J Mol Sci. 2023 Apr 25;24(9):7822. doi: 10.3390/ijms24097822 (PMC10177957; doi:10.3390/ijms24097822)
Supplement: Supplementary file 1 [file ijms-24-07822-s001.zip › ijms-2303876-supplementary.pdf]

Supplementary Material for
A Novel Rabbit Anti-Myoglobin Monoclonal Antibody's Potential Application in Rhabdomyolysis Associated Acute Kidney Injury

Xinyue Wang [1,2,†], Ou Qiao [1,2,†], Lu Han[1,2], Ning Li [1,2,*] and Yanhua Gong[1,2,*]

[1] Institute of Disaster and Emergency Medicine, Medical College, Tianjin University, Tianjin 300072, China; wxy435014@tju.edu.cn (X.W.); qiaoou@tju.edu.cn (O.Q.); stu_hanlu@tju.edu.cn (L.H.)
[2] Tianjin Key Laboratory of Disaster Medicine Technology, Tianjin 300072, China
* Correspondence: lining620@tju.edu.cn (N.L.); gongyanhua@tju.edu.cn (Y.G.).
† These authors contributed equally to this work.

1 Supplementary Figures

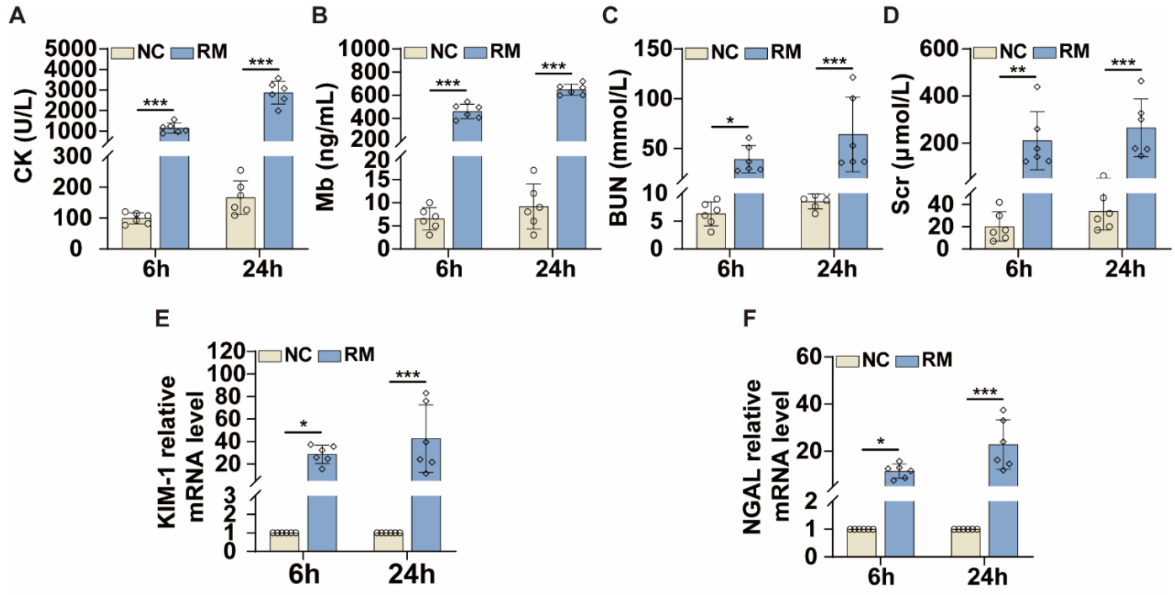

Figure S1. Validation of successful establish the mice RM-AKI model. (A-D) Biochemical indicator detection of CK, Mb, BUN and Scr concentration in the serum. (E-F) qPCR analysis the mRNA expression level changes of KIM-1 and NGAL in the kidney of RM-AKI mice in 6h and 24h, respectively ($n = 6$; *, $p<0.05$; **, $p<0.01$; ***, $p < 0.001$).

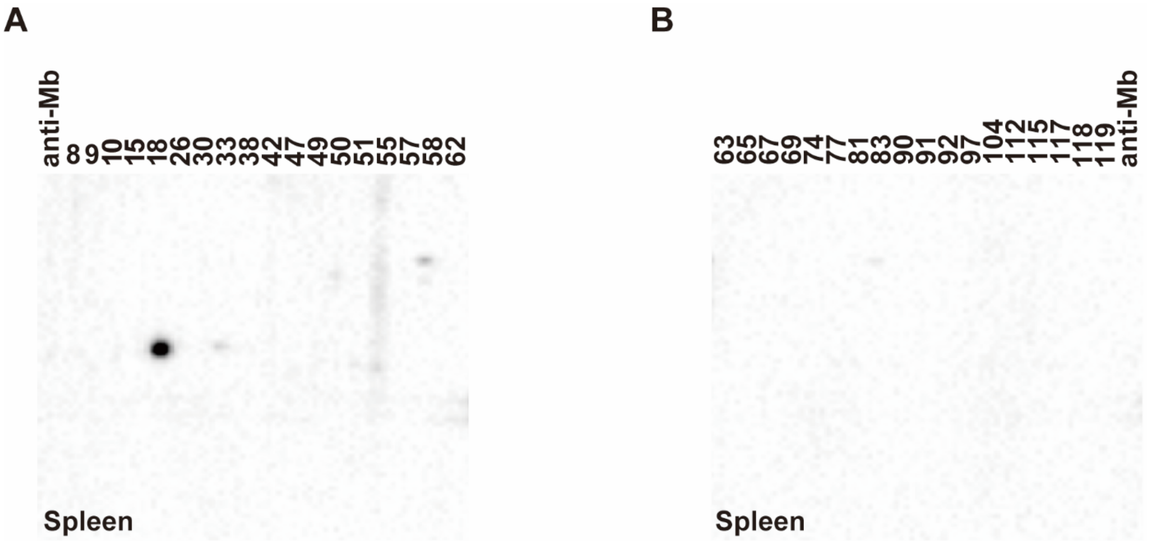

Figure S2. Negative exclude screening of hybridoma-derived rabbit anti-Mb monoclonal antibody. (**A-B**) WB analysis the reactivity of 36 positive hybridoma supernatants (screening from Fig. **3A-D**) with rat spleen tissue protein. Commercial anti-Mb antibody as control.

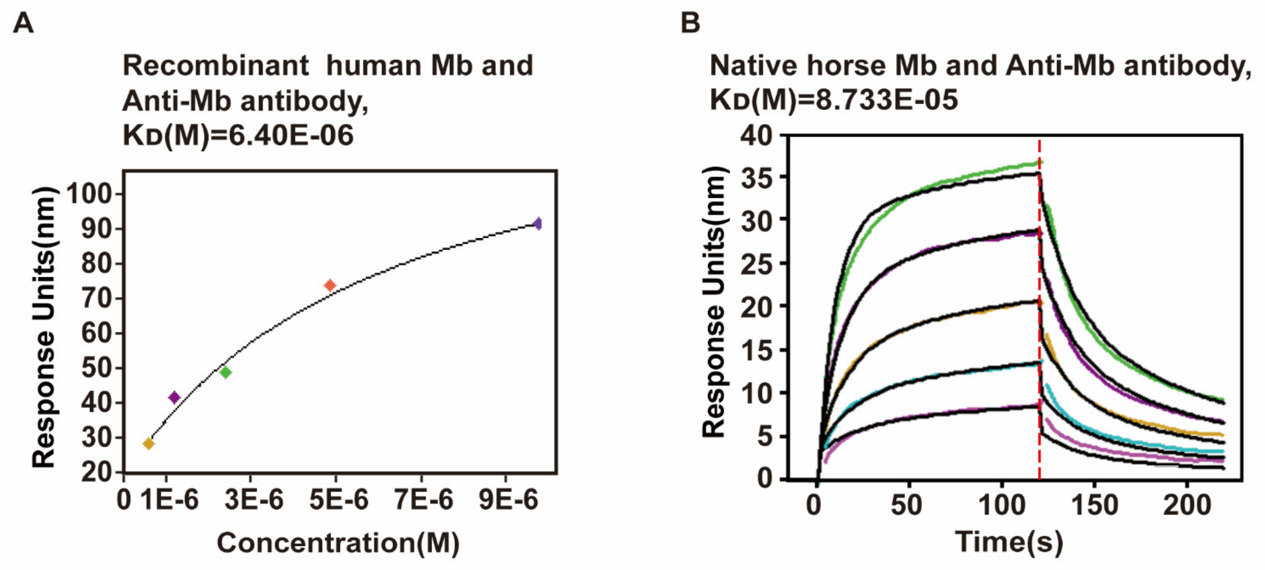

Figure S3. SPR sensorgrams of recombinant anti-Mb monoclonal antibody to the recombinant human Mb protein (**A**) and native horse Mb protein (**B**) immobilized sensor chip. The raw data is shown as a colourful line, the calculated fit is shown as a black line.

2 Supplementary Tables

Table S1. Binding kinetic information form Fortebio detection.

Sample ID	Conc. (nM)	K_D (M)	K_D Error	K_{on}(1/Ms)	K_{on} Error	K_{dis}(1/s)	Full R^2
**Number 9**	534.8	1.60×10^{-8}	4.35×10^{-11}	2.52×10^{4}	5.06×10^{1}	4.04×10^{-4}	0.9979

**Number 42**	534.8	3.44×10^{-8}	1.09×10^{-10}	2.84×10^{4}	8.16×10^{1}	9.75×10^{-4}	0.9983
**Number 49**	534.8	1.36×10^{-7}	1.63×10^{-8}	7.54×10^{4}	8.55×10^{3}	1.02×10^{-2}	0.884
**Number 81**	534.8	5.16×10^{-8}	1.29×10^{-9}	3.46×10^{4}	8.19×10^{2}	1.79×10^{-3}	0.9488
**Number 115**	534.8	4.53×10^{-8}	1.95×10^{-10}	2.78×10^{4}	1.11×10^{2}	1.26×10^{-3}	0.9976
**Number 118**	534.8	2.50×10^{-7}	1.13×10^{-8}	2.45×10^{4}	1.05×10^{3}	6.12×10^{-3}	0.964

Table S2. Binding kinetic information form SPR detection.

Sample	K_D (M)	K_{on} (1/Ms)	K_{off} (1/s)	Rmax (RU)
**H: L=1:1**	5.94×10^{-9}	5.00×10^{4}	2.97×10^{-4}	0.9979
**H: L=1:2**	1.21×10^{-12}	1.66×10^{5}	2.01×10^{-7}	0.0532
**H: L=1:3**	7.12×10^{-10}	1.63×10^{5}	1.16×10^{-4}	0.0473
**H: L=1:4**	1.17×10^{-9}	2.11×10^{5}	2.46×10^{-4}	0.0686

Table S3. The list of primers used in this study.

Gene names	Forward primer (5′-3′)	Reverse primer (5′-3′)
**NGAL**	TGGCCCTGAGTGTCATGTG	CTCTTGTAGCTCATAGATGGTGC
**KIM-1**	GGTCTGTATTGTTGCCGAGTGGAG	GCCTTGTGGTTGTGGGTCTTGTAG
**GAPDH**	AGGTCGGTGTGAACGGATTTG	TGTAGACCATGTAGTTGAGGTCA
